# When Immune Cells Turn Bad—Tumor-Associated Microglia/Macrophages in Glioma

**DOI:** 10.3390/ijms19020436

**Published:** 2018-02-01

**Authors:** Saskia Roesch, Carmen Rapp, Steffen Dettling, Christel Herold-Mende

**Affiliations:** Division of Experimental Neurosurgery, Department of Neurosurgery, University Hospital Heidelberg, INF400, 69120 Heidelberg, Germany; Saskia.Roesch@med.uni-heidelberg.de (S.R.); Carmen.Rapp@med.uni-heidelberg.de (C.R.); Steffen.Dettling@med.uni-heidelberg.de (S.D.)

**Keywords:** microglia, glioma-associated microglia/macrophages, GAMs, glioma, glioblastoma, GBM, tumor microenvironment, TME

## Abstract

As a substantial part of the brain tumor microenvironment (TME), glioma-associated microglia/macrophages (GAMs) have an emerging role in tumor progression and in controlling anti-tumor immune responses. We review challenges and improvements of cell models and highlight the contribution of this highly plastic cell population to an immunosuppressive TME, besides their well-known functional role regarding glioma cell invasion and angiogenesis. Finally, we summarize first therapeutic interventions to target GAMs and their effect on the immunobiology of gliomas, focusing on their interaction with T cells.

## 1. Introduction

High-grade gliomas and especially glioblastoma (GBM) are some of the most aggressive tumors in humans. Despite multimodal therapeutic interventions, the median survival of GBM patients is still restricted to about 15 months [1]. One of the main reasons for the observed treatment resistance and concomitant tumor recurrence is the invasive growth of GBM preventing a total tumor resection as well as profound changes in the tumor microenvironment (TME) [2,3]. Like most extracranial tumors, the TME of brain cancers consists of a substantial proportion of non-neoplastic cells. Hence, the brain hosts some exclusively tissue-resident cell types, including neurons, astrocytes, and microglia as well as a specific brain vasculature as part of the blood-brain barrier [4]. Upon inflammation, such as autoimmune, neurodegenerative, and epileptic disorders as well as brain malignancies, microglia cells are activated to restore the brain homeostasis [5]. This process is further supported by bone marrow-derived macrophages (BMDMs) which also can infiltrate the brain. In malignant gliomas, the mixture of activated brain-resident microglia and BMDMs can comprise up to one third of the tumor mass [6] and collectively act to promote tumorigenesis (pro-tumorigenic) and to create an immunosuppressive TME [7,8]. Accordingly, targeting these tumor-supportive cell types represents a novel promising treatment approach to improve the survival of GBM patients [9].

In the following sections, we will describe the physiological functions of microglia and macrophages, changes occurring in the TME, their impact on anti-tumor immune responses, and how this can be exploited in a therapeutic setting.

## 2. Origin and Physiological Function of Microglia and Macrophages of the Central Nervous System 

Microglia cells are crucial immune cells of the central nervous system (CNS) and serve as tissue-resident macrophages of the brain [10]. They have a considerable influence on brain development and the homeostasis of the neural environment by the phagocytosis of apoptotic cells and by supporting neurogenesis, synaptic refinement, and axonal growth [11,12,13]. Moreover, microglia cells are involved in immune surveillance and are a substantial component of the first line of defense, as described in more detail later [14].

**Figure 1:**
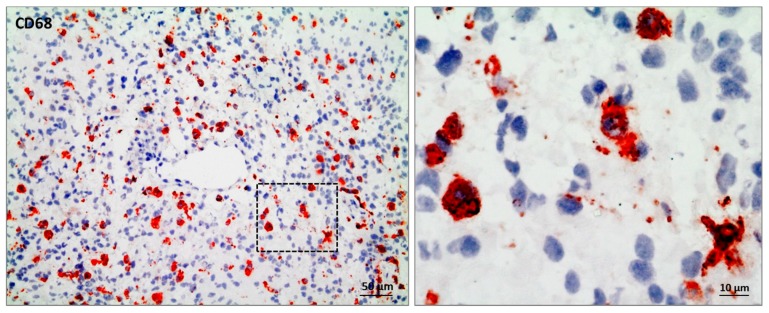
Immunohistochemical staining of CD68 to visualize glioma-associated microglia/macrophages in primary glioblastoma tissue of GBM patient NCH3242. Scale bar 50 µm, magnification 10 µm.

Until recently, it was assumed that microglia cells originated from hematopoietic stem cells of the bone marrow. Nowadays, it has become clear that microglia cells arise from hematopoietic precursor cells of the yolk sac in the early embryogenesis between embryonic day E7.0 and E9.0 [11,15]. In contrast, brain-infiltrating BMDMs originate from hematopoietic stem cells [16,17]. According to their localization, BMDMs can be subdivided into meningeal macrophages, choroid plexus macrophages, perivascular macrophages, and dendritic cells [18,19]. Thus, microglia and BMDMs represent two ontogenetically distinct myeloid cell populations with similar immune regulatory features and the expression of several common markers, such as CD11b, CD68 (Figure 1), ionized calcium-binding adapter molecule 1 (IBA1), CX3C chemokine receptor 1 (CX3CR1), and F4/80 (mouse specific) [5,20,21,22].

In gliomas, the infiltrating BMDM population intermingles with tissue-resident microglia. As a consequence, they are virtually undistinguishable and exceedingly difficult to study independent from one another [7]. Traditionally, microglia are defined as CD11b^+^/CD45^low^ (or CD45^int^), whereas the CD11b^+^/CD45^high^ population accounts for other CNS macrophages [23]. However, upon inflammation microglia can rapidly upregulate CD45 expression, resulting in an incorrect classification as BMDMs [24]. Moreover, the discrimination between microglia and BMDMs relies on the quantitative expression of surface markers as usually assessed by flow cytometry analysis, and therefore is restricted to cell isolates. Recently, the transmembrane protein 119 (TMEM119) was reported as a novel microglia-specific marker to reliably discriminate CNS-resident microglia from BMDMs in both humans and mice [20,25]. However, further studies are needed to verify these findings.

## 3. Microglia Models for Functional Studies

While peripheral macrophages can be easily isolated from blood samples, the purification of brain-resident microglia/macrophages for subsequent functional studies is still much more challenging. Microglia—especially in the context of brain malignancies—are still under-investigated, primarily due to the lack of robust in vitro and in vivo models. For decades, the most common approach to separate human microglia from other CNS cells, such as astrocytes, oligodendrocytes, and neurons, has been based on their strong adherence to plastic vessels [26,27,28]. However, the low yields limit their usage for functional studies. To overcome this limitation the first immortalized murine (BV-2) and human (HMO6) microglia cell lines were generated by retroviral transfection of the *Myc* oncogene in the 1990s [29]. Later on, the commonly used human microglia cell line CHME was transformed with a lentiviral vector expressing the SV40 large T antigen [30,31]. Although the majority of knowledge about microglia biology is provided by such cell models [32], comparative analysis of primary versus BV-2 microglia raised doubts regarding the unrestricted usability of such a model system [33,34]. The authors demonstrated that primary brain-derived microglia cells differ from the immortalized BV-2 microglia cell line in their pathogen-derived lipopolysaccharides (LPS)-induced cytokine, chemokine, and NO expression [34]. More recently, Das et al. performed transcriptome sequencing and observed a microglial gene expression signature including the expression of several transcription factors and epigenetic regulators in primary microglia rather than in BV-2 microglia cells [33]. Further technical progress in this regard was the sorting of microglia/macrophages with the help of magnetic CD11b microbeads, which substantially enhanced the yield and purity of isolated microglia/macrophages for functional studies [35,36].

Another promising approach is the in vitro generation of microglia from embryonic stem cells by using a modified five-step method. This includes (1) isolation and expansion; (2) generation of embryonic bodies by hanging drop cultures; (3) selection and expansion of nestin-positive cells; (4) induction of differentiation through distinct medium and matrix conditions, and finally (5) expansion of the resulting microglia cells [37]. However, due to ethical restrictions a transfer of this knowledge to human microglia might still be difficult. Instead of that a possibility to circumvent the shortcoming of this approach could be the use of human-induced pluripotent stem cells (hiPSC) [38].

Finally, murine lineage tracing models have been employed to distinguish between tissue-resident microglia and BMDMs and to study the fate of these cells in their natural environment [3,24]. The most prominent model in this regard is a CX3CR1^GFP^ knock-in mouse [39]. Moreover, head-protected irradiation (HPI) was used to prevent the migration of peripheral immune cells (for example, BMDMs) into the brain, and thus to solely focus on brain-resident microglia in functional studies [40,41]. Results obtained by this method suggested that intratumoral myeloid cells are mainly composed of tissue-resident microglia, rather than peripheral macrophages [24]. However, such sophisticated models are expensive and not applicable to study human microglia. To circumvent this shortcoming, in vivo/ex vivo organotypic brain slices recapitulating the human in vivo situation and thus enabling scientists to study myeloid cells in their complex environment are increasingly used [42]. For instance, this methodology has been employed using clodronate-filled liposomes to specifically deplete the microglia/macrophage population, and thus to study their influence on tumor cell invasion and the immune microenvironment [43]. Altogether, organotypic brain slices hold promise for studying changes in the myeloid compartment caused by varying mutational loads as well as epigenetic and transcriptional changes of glioma cells in a patient-specific human setting.

## 4. Activation and Polarization of Resting Microglia and Macrophages

Naïve so-called resting microglia cells are extremely sessile and constantly screen their microenvironment with their highly motile pseudopodial extensions [44,45,46,47]. Various pathological events in the CNS such as injuries, viral and bacterial infections, or tissue damage lead to the activation of tissue-resident microglia cells [13]. Upon this type of activation, microglia respond with the expression of co-stimulatory molecules (CD40, CD80, and CD86) and high expression levels of major histocompatibility complex (MHC) II molecules [48,49,50,51,52,53], and can thereby serve as antigen-presenting cells [54]. Thus, microglia can act as a direct link between the innate and adaptive immune system. Moreover, they express the pro-inflammatory tumor necrosis factor (TNF)-α [55], which facilitates the infiltration of macrophages from the periphery [52,56]. Subsequently, the whole myeloid cell population together causes an acute inflammatory response [57,58]. In detail, microglia/macrophages primarily recognize immunogenic antigens, such as LPS via several immune pattern recognition receptors, including toll-like receptors (TLRs), nucleotide-binding oligomerization domain-(NOD)-like receptors, and scavenger receptors (SRs), followed by pathogen clearance through phagocytosis [45,46,47]. LPS stimulation also leads to nuclear factor kappa-light-chain-enhancer of activated B cells (NF-κB) and signal transducer and activator of transcription (STAT)1 signaling, resulting in the expression of pro-inflammatory cytokines, such as interleukin (IL)-1α, -1β, -6, -12, -23, and chemokines, such as CC-chemokine ligand (CCL)2–5 and CCL8–11 [52,59]. Under these circumstances, microglia/macrophages also express redox molecules (NADPH oxidase, phagocytic oxidase), SRs, and produce high levels of inducible nitric oxide synthase (iNOS) for nitric oxide production. This metabolic state has often been described as a characteristic feature of the pro-inflammatory, M1-like activated microglia/macrophage phenotype [52,60]. Importantly, microglia/macrophage activation is a highly dynamic process. The induction of an inflammatory response by M1-like microglia/macrophages is tightly regulated through a subsequent polarity transition to an anti-inflammatory M2-like phenotype. This results in the downregulation of immune responses, prevents tissue damaging, and supports healing processes by the secretion of a series of anti-inflammatory and immune-regulating factors [61,62]. Besides general microglia/macrophage markers (CD68, CD11b, IBA1), M2-polarized microglia/macrophages are characterized by the co-expression of several markers such as CD163, CD204, and CD206 [63]. Mantovani et al. introduced three different M2 subtypes (M2a, M2b, M2c) which evolve upon different environmental signals and exert distinct functions [64]. While M2a and M2b microglia/macrophages are both involved in immune regulatory functions, the M2c phenotype instead contributes to an attenuation of inflammatory responses and tissue remodeling, and when occurring in neoplastic conditions considerably promotes tumor growth [64]. In more detail, M2a polarization is mainly caused by stimulation through T helper cell (T_H_)-derived IL-4 and IL-13 [65]. Binding of the IL-4 receptor (IL-4R) activates the transcription factor STAT6 which induces the expression of an anti-inflammatory cytokine/chemokine signature, including transforming growth factor (TGF)-β, IL-2 receptor α (IL-2RA), and CCL15, -17, -22, and -24 [59,66,67]. Simultaneously, IL-4R signaling leads to a silencing of the M1-characteristic NF-κB signaling [52]. In comparison to M2a microglia/macrophages, the M2b subtype is triggered by immune complexes, TLRs, or IL-1R antagonists [64]. Downstream signaling of these receptors induces the secretion of IL-1, IL-6, IL-10, CCL1, and TNF-α, which mediates immune regulation through T_H_2 and regulatory T cell (T_reg_) activation.

**Figure 2:**
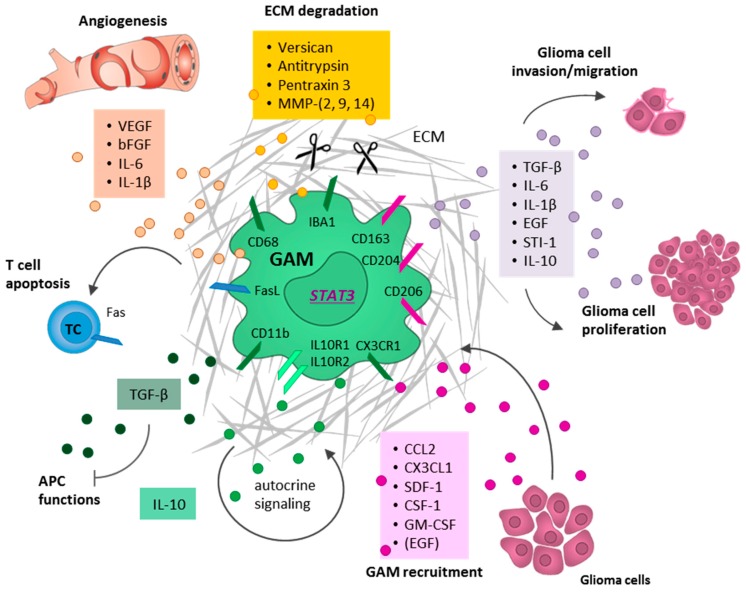
Contribution of glioma-associated microglia/macrophages (GAMs) to a pro-tumorigenic tumor microenvironment (TME). Glioma-associated microglia/macrophages (GAMs) are recruited to the tumor lesion by several glioma cell-derived factors (pink: CCL2, CX3CL1, SDF-1, CSF-1, GM-CSF, GDNF, EGF), resulting in a polarization towards an anti-inflammatory and pro-tumorigenic M2-like phenotype. In the presence of glioma cells, GAMs express the IL-10 receptor and the cytokine itself. Therefore, the pro-tumorigenic M2-like phenotype can be sustained by autocrine IL-10 signaling. To study M2-like polarized GAMs, a combination of microglia/macrophage markers in general (green: CD11b, CD68, IBA1, and CX3CR1) and more M2-like specific markers (pink: CD163, CD204, CD206, and STAT3) are employed. Moreover, GAMs are endowed with a M2-associated secretome facilitating extracellular matrix (ECM) degradation (yellow: versican, antitrypsin, pentraxin 3, and several matrix metalloproteinases (MMPs)), and angiogenesis (red: VEGF, bFGF, IL-6, and IL-1β). Through the secretion of TGF-β, IL-6, IL-1β, EGF, STI-1, and IL-10 (violet), GAMs actively promote glioma cell proliferation, facilitate their invasion and migration, and impair immune cell functions.

Finally, the activation to the M2c phenotype is triggered by the exposure to anti-inflammatory cytokines secreted for instance by tumor cells, such as IL-10, TGF-β, and glucocorticoids. This M2c phenotype is often referred to as acquired deactivated microglia/macrophages and seems to be the predominant phenotype in the context of brain malignancies (Figure 2) [49,64,68,69,70].

As a response to IL-10, the transcription factor STAT3 is phosphorylated and induces the transcription of TGF-β, found in inflammatory zone 1 (FIZZ1), and peroxisome proliferator-activated receptor (PPAR)-γ as well as an autocrine stimulation, leading to an anti-inflammatory microenvironment [52]. Moreover, the M2c phenotype facilitates ECM deposition and tissue remodeling by the expression of versican, antitrypsin, and pentraxin 3 [71,72].

It is noteworthy that the distinction between pro-inflammatory M1-polarized and anti-inflammatory M2-polarized GAMs is difficult to study due to the lack of unique markers to assess their activation phenotype in the continuous process of GAM polarization. Nevertheless, M1-polarized GAMs have often been associated with the expression of CD40, CD74, and MHC II, whereas the expression of CD163, CD204, CD206, arginase 1 (ARG1), FIZZ1, and phosphorylated STAT3 (pSTAT3) has been attributed to more M2-polarized GAMs [52]. In gliomas, the analysis of GAMs revealed a predominant population of CD163^+^ and CD204^+^ anti-inflammatory M2 GAMs in association with higher tumor grade and a worse patient survival [73]. Moreover, Komohara et al. demonstrated that glioma cell-derived factors, such as TGF-β and macrophage colony-stimulating factor (M-CSF; alternative name colony-stimulating factor 1 (CSF-1)), promote the upregulation of several M2 markers (CD163 and CD204), and thereby actively contribute to the M2-like GAM polarization [73].

In summary, depending on the respective stimuli, the activation of microglia/macrophages is either directed towards the classical, pro-inflammatory more M1-like phenotype or towards the alternatively activated, anti-inflammatory more M2-like phenotype [53]. Although recent data suggest an even more complex situation [74], the M1/M2 paradigm is a rationalized model representing the two opposing roles of microglia/macrophages and provides a useful framework for further characterization, even in the context of brain malignancies [75]. Commonly used markers to experimentally distinguish between BMDMs and tissue-resident microglia as well as between the different polarization states are summarized in Table 1. In the following section, the comprehensive influence of tumor cells on the polarization of GAMs and its consequences for immune responses and tumor growth will be discussed in more detail.

## 5. Microglia/Macrophages—Glioma Cell Crosstalk

GAMs intimately interact and co-evolve with malignant tumor cells rather than being only a bystander. After the active tumor cell-mediated recruitment and polarization into the pro-tumorigenic M2-like phenotype, GAMs substantially contribute to tumor growth, tumor cell migration, and invasion. Moreover, GAMs facilitate the destruction of the ECM, foster neoangiogenesis, and contribute massively to an immunosuppressive microenvironment [7].

### 5.1. Glioma Cells Actively Recruit GAMs and Induce a M2-Like Polarization

One of the most important chemoattractants known to recruit GAMs is the monocyte chemoattractant protein (MCP)-1 (alternative name: C-C motif ligand 2 (CCL2)), which is secreted by astrocytoma and glioblastoma cells in vitro and in vivo [88,91]. In vitro chemoattraction could successfully be inhibited by an MCP-1-neutralizing antibody [91]. In vivo mouse experiments demonstrated that glioma cell-derived MCP-1 increases GAM infiltration [92]. Moreover, the extent of MCP-1 expression is associated with glioma grade [93], and promotes neoangiogenesis, tumor cell proliferation, and invasion [88] as well as the infiltration of T_regs_ [94]. However, other reports claim that GAM infiltration depends on the expression of MCP-3 rather than MCP-1 [95]. In a murine astrocytoma model, it could further be shown that the secretion of the stroma-derived factor (SDF)-1 (alternative name: CXC motif chemokine (CXCL)12) by glioma cells specifically promotes the intratumoral accumulation of GAMs in normoxic rather than hypoxic tumor areas [96]. Glioma cells also release M-CSF (CSF-1), which markedly promotes GAM motility and converts microglia into the pro-tumorigenic M2-like phenotype [97]. In addition, Sielska et al. demonstrated that the cytokine granulocyte-macrophage colony-stimulating factor (GM-CSF) is also secreted by glioma cells and induces GAM invasion in vitro [98]. They further showed in an organotypical brain slice model, in which GM-CSF knockdown in GL261 glioma cells reduced the GAM-dependent invasion. Moreover, depleting GM-CSF in a murine astrocytoma model also resulted in a reduced infiltration of IBA1^+^ GAMs [98].

Furthermore, the epidermal growth factor (EGF) has been identified as a paracrine motility factor directing microglia to the lesion site [99]. The inhibition of EGF receptor (EGFR)/mitogen-activated protein kinase (MAPK) signaling in microglia reduces the production of the pro-inflammatory cytokines IL-1β and TNF-α, as shown in an in vivo rodent model [100]. Accordingly, Qu et al. demonstrated that LPS mediates EGFR phosphorylation and thereby activates the extracellular-signal regulated kinase (ERK) signaling cascade in microglia cells. ERK inhibition by U0126 significantly attenuated the LPS- and EGF-induced migration of microglia in a BV-2 culture model. Based on these findings, the authors suggested further studies to verify the inhibition of ERK as an additional therapeutic approach to target microglia in GBM [101].

**Figure 3:**
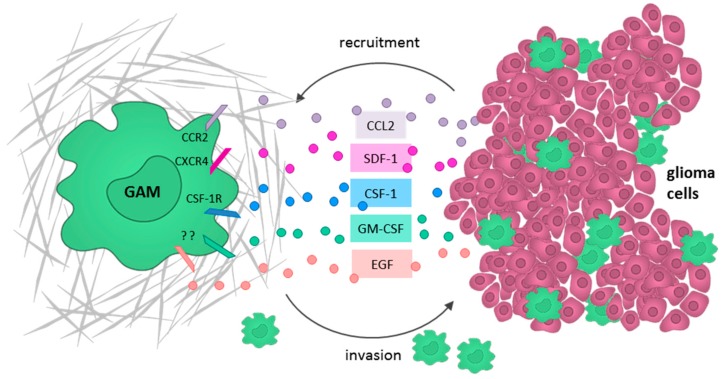
Recruitment of glioma-associated microglia/macrophages (GAMs) by glioma cells through the secretion of soluble factors, such as CCL2 (violet), SDF-1 (pink), CSF-1 (blue), GM-CSF (green), and EGF (red).

In summary, recruitment and subsequent M2 polarization of GAMs has been shown to be mediated by multiple glioma cell-derived chemoattractants such as MCP-1 (CCL2), SDF-1 (CXCL12), M-CSF (CSF-1), GM-CSF, and EGF (Figure 3). Thus, the inhibition of these ligands or the corresponding receptors should be considered as future therapeutic targets.

### 5.2. GAMs Promote Glioma Cell Invasion and Tumor Growth

In 2002, Bettinger et al. observed a threefold increased migration of glioma cells when exposed to microglia isolated from murine brains [102]. The authors hypothesized that this effect was mediated by the release of microglia-derived chemoattractants into the medium [102]. Meanwhile, several GAM-derived factors such as TGF-β, stress-inducible protein (STI)-1, IL-6, IL-1β, and EGF have been identified as promoters of glioma cell invasion [7].

Among these, the TGF-β superfamily members 1-3 have been extensively studied and characterized as immunosuppressive cytokines, which are upregulated in glioma tissues and secreted by glioma cells [103,104,105]. In particular, GAM-derived TGF-β2 induces the expression of MMP-2, an enzyme which promotes ECM deposition and thus facilitates the invasive properties of glioma cells in vitro [106]. Additionally, MMP-2 expression is significantly associated with aggressiveness of astrocytoma and an unfavorable prognosis of GBM patients [107]. Furthermore, the expression of membrane type 1-matrix metalloproteinase 1 (MT1-MMP), which is necessary to activate pro-MMP-2, increases with glioma grade [108]. Hence, the antibody-targeted inhibition of MT1-MMP impaired glioma growth [109].

The co-chaperone STI-1 is another GAM-derived chemoattractant driving glioma cell proliferation and migration in vitro. In addition, it has been shown that the expression of STI-1 by GAMs increases with tumor grade, while the STI-1 expression level in circulating blood monocytes remains unchanged [110].

As mentioned earlier, CSF-1 (M-CSF) secreted by glioma cells facilitates recruitment and M2-like activation of GAMs [97]. Additionally, in a transgenic mouse model, De et al. demonstrated that CSF-1 overexpression increases the density of GAMs in high-grade gliomas [111].

Further, there is an ongoing discussion about the role of EGF as a promoter of tumor cell invasion and a modulator of microglial motility [99]. For instance, an increased concentration of secreted EGF was detected neither in GAM-derived supernatants nor in protein lysates [112]. However, it has been shown that microglia cells also express the membrane-spanning EGF precursor [112] which might also be capable of activating EGFR signaling in glioblastoma cells in a contact-dependent manner. This is of particular importance because EGFR is overexpressed or amplified in about 60% of primary glioblastoma and characteristic for a highly aggressive phenotype [113,114].

### 5.3. GAMs Affect Neoangiogenesis by Destructing the Extracellular Matrix

Several studies revealed that GAMs facilitate the vascularization of brain tumors by secreting high levels of pro-angiogenic factors such as vascular endothelial growth factor (VEGF) [115]. Accordingly, VEGF receptor (VEGFR) blockage by the administration of Sunitinib (Sutent^®^) combined with the VEGF inhibitor Bevacizumab (Avastin^®^) resulted in a reduction of myeloid infiltrates, decreased tumor vascularity, and prolonged survival in a GBM mouse model [116].

Like VEGF, IL-6 facilitates tumor vascularization and promotes tumor growth [117]. IL-6 expression is mainly induced by the activation of the receptor for advanced glycation end products (RAGE) [118]. Correspondingly, RAGE knockout in murine microglia resulted in a reduced IL-6 (and also VEGF) expression and abrogated angiogenesis. However, after an injection of macrophages from healthy donor mice without RAGE knockout, a normalized tumor vasculature was restored [119]. Further evidence for a contribution of GAMs to neoangiogenesis came from GAM (CD11b^+^) depletion experiments, leading to a strongly decreased vessel density and a reduced tumor volume after depletion [115]. Interestingly, a selective depletion of microglia cells (using bone-marrow chimeras in combination with HPI) led to comparable results, indicating that resident microglia rather than peripheral macrophages promote the vascularization of brain tumors [115].

By employing time lapse in vivo microscopy in a murine glioma model, Bayerl et al. identified four major GAM populations based on parameters such as cell shape, cell volume, cell velocity, and track displacement: the ‘resting’, ‘phagocytic’, ‘interacting’, and ‘mobile’ phenotype [45]. Interestingly, GAMs accumulating in the perivascular niche showed the highest motility and thus most likely reflect the mobile phenotype. This further suggests an increased interaction of GAMs with endothelial cells and pericytes [45]. However, to elucidate the functional properties of these different GAM phenotypes, and thus to study their influence on other cell types within the TME, the identification of specific markers or marker combinations is warranted.

### 5.4. GAMs Contribute to an Immunosuppressive TME

M2-polarized GAMs exert their immunosuppressive functions by soluble factors as well as by direct cell-cell interactions. First the focus is on the effect of GAM-secreted cytokines on different immune cell types and then the influence of direct cell-cell contact of GAMs and other immune cells will be discussed.

In glioma tissues, only low amounts of the M1-associated pro-inflammatory cytokines IFN-γ, TNF-α, IL-2, and IL-12 were detected [88,120], while high amounts of M2-associated anti-inflammatory cytokines such as TGF-β, IL-6, and IL-10were found in these tumors, indicating an immunosuppressive TME [88,120]. Further investigations identified GAMs as well as glioma cells as the main source of these cytokines [121,122]. Interestingly, in vitro microglia monocultures only secreted low amounts of TGF-β. However, co-culture with glioma cells led to an increased TGF-β secretion [123]. The resulting TGF-β signaling has been shown to induce the downregulation of MHC II molecules as well as the co-stimulatory molecules CD80 (B7-1) and CD86 (B7-2) in GAMs, leading to a reduced phagocytic activity [124,125,126]. In contrast, the expression of B7 molecules as well as MHC II increased in isolated GAMs in the absence of tumor cells [127]. The assumption that more M1-like microglia/macrophages contribute to a less immunosuppressive TME is supported by the observation of a positive correlation between the expression of MHC II and B7 molecules on GAMs and the amount of tumor-infiltrating lymphocytes (TILs), as assessed in a rodent glioma model [127].

Similar to TGF-β, IL-10 also attracts a lot of interest for its contribution to an immunosuppressive TME through the inhibition of antigen-presenting cells, T cell proliferation, and through the induction of T_regs_ [128,129,130]. Furthermore, increased IL-10 expression levels are associated with glioma malignancy [131]. GAMs are regarded as the main source of IL-10 in human GBM [121]. The transcription of IL-10 in GAMs is mainly based on STAT3 signaling, which has been shown to be enhanced in tumor-derived GAMs as compared to normal microglia/macrophages [126,129]. In line with these observations, STAT3 blockage in GAMs led to a decreased secretion of IL-10 [129] and a variety of other inflammatory molecules, such as IL-4, IL-6, IL-11, and IL-23, as well as the growth factors EGF, platelet-derived growth factor (PDGF), hepatocyte growth factor (HGF), and fibroblast growth factor (FGF), which might contribute to the formation of an immunosuppressive TME [132]. Moreover, activated pSTAT3 expression has been shown to be associated with an increased tumor grade and a worse survival of glioma patients, whereby no expression was detected in normal brain and low-grade astrocytoma [87,133,134,135], suggesting pSTAT3 as a negative prognostic factor [87]. In addition, GAMs isolated from freshly resected GBM specimens revealed increased pSTAT3 levels [126]. Furthermore, it has been demonstrated that glioma cell-derived conditioned medium is able to increase STAT3 activity in microglia cells, leading to increased secretion of the anti-inflammatory M2-like cytokines IL-6 and IL-10 [129]. A siRNA-mediated STAT3 inhibition reversed this M2-like cytokine expression profile. Additionally, inactivation of STAT3 in intracranial GL261 tumors by siRNA resulted in GAM activation and tumor growth inhibition [129]. Taken together, these results suggest STAT3 targeting as a promising treatment approach, which will be further elaborated upon later on [136,137,138].

As introduced before, not only GAM-secreted cytokines but also direct cell-cell interactions of GAMs with other cell types exert immunosuppressive properties. For example, it has been described that almost all GAMs express the membrane-bound Fas ligand (FasL) [139]. Considering the fact that apoptotic T cells are characterized by the expression of the Fas receptor, GAMs are supposed to drive apoptosis of activated T cells through Fas-FasL interaction, and thereby further enhance an immunosuppressive TME [140,141]. Consistently, the inhibition of FasL in gliomas resulted in a threefold increased infiltration of TILs, highlighting a potential role of GAMs for immune evasion [139].

Providing evidence for a direct interaction with T_regs_, studies on autoimmune encephalomyelitis demonstrated that M2-like polarized microglia (MHC II^+^/CD40^dim^/CD86^dim^/IL-10^+^) were able to induce antigen-specific T_regs_ in vitro (CD4^+^/forkhead box P3 (FOXP3^+^)) [142]. These T_regs_ inhibited the proliferation of effector T cells, underlining the regulatory role of microglia even for adaptive immune responses. Further studies are needed to unravel a similar T cell-microglia crosstalk in the context of brain malignancies.

## 6. Targeting GAMs to Reinforce the Anti-Tumor Immunity

Preclinical studies targeting M2-polarized and thus immunosuppressive GAMs revealed promising results [143,144,145,146]. Therefore, it has been assumed that immunotherapeutic interventions might benefit from additional GAM-directed treatments to improve the prognosis of glioma patients [9]. Possible approaches aiming either at blocking chemoattractant receptors/ligands to reduce GAM recruitment and invasion, or fostering depletion or re-polarization of pro-tumorigenic, anti-inflammatory M2-like GAMs to enrich the pro-tumorigenic M1-like GAMs will be discussed in the following. Table 2 summarizes therapeutic drugs targeting microglia. 

As mentioned earlier in this review, the colony-stimulating factor 1 receptor (CSF-1R) ligand CSF-1 (M-CSF) is secreted by glioma cells and facilitates the recruitment and M2 polarization of GAMs [97]. Thus, CSF-1R antagonists receive increasing attention as novel therapeutic targets [147]. In a preclinical model, the blockage of CSF-1R signaling in glioma-bearing mice by using the anti-CSF-1R antibody Pexidartinib (PLX3397) resulted in a significantly reduced tumor infiltration of GAMs [112]. They also observed a decreasing tumor volume and significantly increased survival times of treated mice [112]. However, the administration of PLX3397 within a phase II clinical trial to recurrent GBM patients as a single agent therapy did not show efficacy [148]. Future studies are warranted to explore if a combination with other types of treatments such as immunotherapies will be able to improve treatment effects.

The application of another CSF-R1 inhibitor BLZ945 in glioma-bearing mice as a single agent solely revealed a decreased expression of M2 markers rather than depleting pro-tumorigenic GAMs [97]. As a possible explanation, another group recently demonstrated in a murine model that CSF-R1 tolerance occurring after BLZ945 administration might be driven by the elevated activity of insulin-like growth factor (IGF)-1)/phosphatidylinositol 3-kinase (PI3K) signaling [149]. To avoid this type of treatment resistance, BLZ945 was administered in combination with either IGF-1 or PI3K antagonists, leading to a markedly reduced tumor progression and improved survival [3,149].

Besides IGF-1/PI3K signaling, several glioma cell-derived factors, such as GM-CSF and IFN-γ, seem to protect GAMs from CSF-1R inhibitor-mediated depletion and thereby mediate therapy resistance [97].

Similarly, Pradel et al. showed that after the administration of Emactuzumab (RG7155), another therapeutic anti-CSF-1R antibody, glioma cell-derived IL-4 was able to rescue GAM viability and thus counteracted CSF-1R treatment [150]. Furthermore, RG7155-resistant GAMs turned out to be endowed with a substantially increased expression of the M2 marker CD206. This led to the assumption that CD206^hi^ GAMs represent an RG7155-resistant GAM population [150]. Accordingly, it could be shown that patients with a high IL-4 expression most likely do not benefit from CSF-1R-targeting agents [150]. Nevertheless, CSF-R1 blocking strongly reduced the amount of F4/80^+^ microglia/macrophages and was accompanied by an increased CD8^+^/CD4^+^ T cell ratio [151], which again mandates a combinatorial treatment together with a T cell-based therapy. Along this line of reasoning, RG7155 treatment has now been combined with an immune checkpoint inhibitor therapy directed against programmed cell death 1 ligand 1 (PD-L1; Atezolizumab, MPDL3280A) in a recently started phase I study (NCT02323191).

Another approach to target GAMs is the pharmacological inhibition of the SDF-1 receptor (CXCR4) by synthetic peptides, such as AMD3100, peptide R, E5, T140, or LY2510924 [152,153,154,155,156]. As mentioned before, SDF-1 (CXCL12) is a well-known glioma cell-derived factor essential for GAM recruitment [96,157,158]. Thus, receptor blockage aims at reducing GAM recruitment. Currently, two clinical trials (NCT01977677, NCT01339039) are ongoing evaluating the potential and applicability of the SDF-1 inhibitor Plerixafor (AMD3100, Mozobil^®^, #05379530, Sanofi Genzyme, Cambridge, MA, USA) in gliomas [144,159]. Plerixafor was shown to inhibit SDF-mediated chemotaxis of myeloid cells in vitro and has already been approved for the therapy of multiple myeloma and lymphoma [160]. In a preclinical study using U-87 MG intracranial xenografts, CXCR4 receptor blocking through peptide R promotes polarization towards M1-like GAMs and additionally impairs metabolic activity and the proliferation of glioma cells in vitro [144,145].

Besides chemokine receptors, chemoattractants such as CCL2 (MCP-1) can also be blocked and hence employed as therapeutic targets. CCL2 increases the infiltration of GAMs and the CCL2 expression is related to the World Health Organization (WHO) grade of gliomas [92]. With Minocycline (an antibiotic), Telmisartan (an anti-hypertensive drug), and Zoledronic (a bisphosphonate), three non-cytotoxic drugs are known to decrease CCL2 synthesis and thus target pathologically activated monocytes, macrophages, dendritic cells, and microglia cells. All three drugs have already been approved, though only for other applications. Since they are highly brain-penetrant, they will be tested in a clinical trial for the treatment of primary glioblastoma patients [161].

Another approach aims to reverse the tumor-promoting effect of GAMs by skewing them back to a pro-inflammatory M1-like phenotype. Apart from the already mentioned CSF-1R blockage, an additional key target in this regard is the transcription factor STAT3. Currently, the STAT3 inhibitor WP1066 is under investigation in a phase I clinical trial in patients with recurrent glioma or melanoma brain metastasis (NCT01904123). WP1066 prevents the phosphorylation of STAT3 and thus significantly inhibits cell survival and proliferation, as well as VEGF production, as shown in vitro and in vivo using a renal cell carcinoma xenograft model [162]. As already mentioned, STAT3 inhibition activates anti-tumorigenic M1-like GAMs, resulting in glioma growth inhibition, and revealing the induction of glioma cell apoptosis [129,138,163]. It remains to be seen if these promising results obtained from preclinical studies can be confirmed in clinical trials.

## 7. Conclusions

While many efforts have been undertaken concerning the origin, morphological, and functional heterogeneity of microglia/macrophages, rather limited insights have been gained into the complex interaction and dynamics between GAMs and other cells of the TME. Nevertheless, to successfully target GAMs and thus to improve patient survival, a deeper knowledge is needed regarding the plasticity of this cell population. While the vast majority of preclinical analysis focuses on the crosstalk of GAMs and malignant tumor cells, the interaction of GAMs with other immune cells is still poorly understood. However, to successfully target the immunosuppressive M2-like GAM population in a clinical setting, it seems to be indispensable to better understand the highly complex interplay of GAMs and other immune cell types of the innate as well as the adaptive immune system.

## Figures and Tables

**Table 1:** Glioma-associated microglia/macrophage markers.

Marker	Microglia	BMDMs	M1-Like	M2-Like	Species	Reference
CD68	X	x			human/murine	[7,76]
IBA1	X	x			human/murine	[77]
CD11b	X	x			human/murine	[78,79]
F4/80	X	x			murine	[80]
CX3CR1^+^/CCR2^−^	X				human/murine	[81,82]
CX3CR1^−^/CCR2^+^		x			human/murine	[81,82]
CD11b^+^/CD45^low^	X				human/murine	[7,23]
CD11b^+^/CD45^high^		x			human/murine	[7,23]
MHC II^high^			x		human/murine	[83,84]
CD80^high^/CD86^high^			x		human/murine	[84,85]
CD80^low^/CD86^low^				x	human/murine	[84,85]
CD74			x		human	[86]
NF-κB/STAT1			x		human/murine	[52,56]
iNOS/NO			x		human/murine	[52,60]
TMEM119	X				human	[20,25]
pSTAT3				x	human/murine	[73,87]
CD163				x	human/murine	[63,73,88]
CD204				x	human/murine	[89]
CD206				x	human/murine	[65]
FIZZ1					(human)/murine	[6,52,90]
ARG1					murine	[6,52,90]

BMDMs = bone marrow-derived macrophages.

**Table 2:** Therapeutic drugs to target microglia.

Drug Name	Target/Function	Suggested Mode of Action	Study Phase	Tumor Types	Identifier	References
PLX3397 (Pexidartinib)	CSF-1R inhibitor	Reduced GAM infiltration	preclinical	GBM	N/A	[112,164]
PLX3397 (Pexidartinib)	CSF-1R inhibitor	GAM elimination	II and I/IIb	rGBM, pGBM	NCT01349036, NCT01790503	[148]
BLZ945	CSF-1R inhibitor	Inhibition of GAM proliferation, blocking of tumor progression, enhancement of CD8^+^ T cell infiltration	preclinical	GBM	N/A	[97,165]
RG7155 (Emactuzumab)	CSF-1R inhibitor	Alters macrophage polarization and blocks glioma progression	preclinical	GBM	N/A	[97,150]
RG7155 (Emactuzumab)	CSF-1R inhibitor	CSF-R1 inhibition	I	GBM	NCT02323191	[3,149]
Plerixafor (AMD3100)	CXCR4 antagonist	Reduced GAM recruitment by inhibition of chemotaxis	I/II	HGG	NCT01977677, NCT01339039	[144,159,166]
Peptide R	CXCR4 antagonist	M1-like polarization	preclinical	GBM	N/A	[144,145]
MTZ regimen	CCL2 inhibitor	Reduced GAM recruitment by inhibition of chemotaxis	preclinical	GBM	N/A	[161]
WP1066	STAT3 inhibitor	M1-like polarization through STAT3 blocking	I	GBM, glioma	NCT01904123	[137,138,163]

CCL2 = CC chemokine ligand 2; CSF-1R = colony-stimulating factor 1 receptor; CXCR4 = CXC motif chemokine receptor 4 (=SDF-1); GAM = glioma-associated microglia/macrophages; GBM = glioblastoma; HGG = high-grade glioma; MTZ regimen = trimodal non-cytotoxic drugs (minocycline (M), Telmisartan (T), Zoledronic (Z)); pGBM = primary glioblastoma; rGBM = recurrent glioblastoma; STAT3 = signal transducer and activator of transcription 3.
